# Carbon storage in the seagrass meadows of Gazi Bay, Kenya

**DOI:** 10.1371/journal.pone.0177001

**Published:** 2017-05-10

**Authors:** Michael N. Githaiga, James G. Kairo, Linda Gilpin, Mark Huxham

**Affiliations:** 1 Kenya Marine and Fisheries Research Institute, Mombasa, Kenya; 2 School of Applied Sciences, Edinburgh Napier University, Edinburgh, United Kingdom; The University of Hong Kong, HONG KONG

## Abstract

Vegetated marine habitats are globally important carbon sinks, making a significant contribution towards mitigating climate change, and they provide a wide range of other ecosystem services. However, large gaps in knowledge remain, particularly for seagrass meadows in Africa. The present study estimated biomass and sediment organic carbon (C_org_) stocks of four dominant seagrass species in Gazi Bay, Kenya. It compared sediment C_org_ between seagrass areas in vegetated and un-vegetated ‘controls’, using the naturally patchy occurence of seagrass at this site to test the impacts of seagrass growth on sediment C_org._ It also explored relationships between the sediment and above-ground C_org,_ as well as between the total biomass and above-ground parameters. Sediment C_org_ was significantly different between species, range: 160.7–233.8 Mg C ha^-1^ (compared to the global range of 115.3 to 829.2 Mg C ha^-1^). Vegetated areas in all species had significantly higher sediment C_org_ compared with un-vegetated controls; the presence of seagrass increased C_org_ by 4–6 times. Biomass carbon differed significantly between species with means ranging between 4.8–7.1 Mg C ha^-1^ compared to the global range of 2.5–7.3 Mg C ha^-1^. To our knowledge, these are among the first results on seagrass sediment C_org_ to be reported from African seagrass beds; and contribute towards our understanding of the role of seagrass in global carbon dynamics.

## Introduction

Carbon sinks in terrestrial ecosystems are better studied than those in marine plant communities. However, the global importance of vegetated coastal habitats as carbon sinks has become appreciated over the last decade [1]. These ‘blue carbon’ ecosystems (tidal marshes, mangroves and seagrass meadows) have recently been demonstrated to capture and store huge stocks of carbon, and their management and conservation may play an important part in global climate change mitigation strategies [2,3,4,5]. Although they occupy less than 2% of the world’s ocean surface area [6], blue carbon ecosystems are estimated to bury nearly 27.4 Tg C yr^-1^ which is about 10% of the yearly estimated organic carbon (C_org_) burial in the oceans [7]. Unlike many terrestrial systems that store C_org_ primarily in living biomass, vegetated coastal ecosystems store much of their C_org_ stock in the sediment, which may produce carbon sinks of hundreds to thousands of years age [7]. However, this stored C_org_ risks being released back to the atmosphere when blue carbon ecosystems are degraded [7].

Seagrass meadows are the most extensive of the blue carbon ecosystems, with an estimated global surface area of between 300,000 to 600,000 km^2^ [1,7]. Despite this wide spatial coverage seagrasses are the least well-studied blue carbon ecosystem. They provide important ecosystem services that include: support for commercial fisheries, worth $ 3500 ha^-1^ yr^-1^ [8], and subsistence fisheries [9], sediment stabilization [10], improved water quality and light availability [11,12] and nutrient cycling (estimated to be worth $ 3.8 trillion yr^-1^ globally [13]. In addition, seagrasses are recognized as one of the most efficient carbon sinks on the planet [3]. Seagrass meadows store about twice as much C_org_ per unit area as soils in productive temperate and upland tropical forests, and provide a global carbon sink of approximately 19.9 Pg [3,14]. This is approximilately equal to the combined amount of C_org_ stored in the world’s tidal marshes and mangrove forests which is estimated at 10 Pg [15]. An estimated 50% of the C_org_ buried in seagrass meadows’ sediment is thought to be of external origin [4]. The dense canopies of seagrasses reduce water flow velocity, thus promoting the trapping and deposition of sediment and particles from the water column [16,17]. Analysis of a global data set revealed that the mean seagrass biomass C_org_ was 2.52 ± 0.48 Mg C_org_ ha^-1^ (±95% CI), while sediment C_org_ was estimated to account for between 0.002–48% of the sediment dry weight [3]. However, these findings may be inaccurate considering the uneven distribution of research into seagrass carbon budgets globally and the large variation recorded between different sites. Much of the information on seagrasses, especially on sediment C_org,_ is from Mediterranean and Australian seagrass beds [1,2,7]. Past global reviews of seagrass carbon acknowledged the disproportionately low contribution of data from the African continent [3,18]. A recent review of seagrass biomass and productivity in Africa found no published estimates of seagrass sediment carbon from the continent [19]. Seagrass beds are suffering rapid global decline; almost a third of all seagrass areas are thought to have been lost in the last 140 years [20] and current rates of loss are estimated at around 1.5% year^-1^ [21]. This implies that a significant amount of the stored C_org_ could soon be remineralized and that the potential for future carbon capture is being diminished, undermining efforts to mitigate climate change [3]. However, the amount of carbon that could be remineralised at a global scale remains highly uncertain. This is because: first, the vulnerability of this stored carbon to ecosystem change and degradation is little studied (with one recent paper showing surprising persistence of buried carbon following seagrass removal [14]. Second, the huge gaps in knowledge on the extent and quantity of the global seagrass carbon sink make global estimates very imprecise. Improving our knowledge of carbon storage and burial rates in seagrass ecosystems, of how these variables differ between sites and of the controls on burial rates and sink sizes is fundamental in achieving a better understanding of how seagrass meadows may contribute to slowing global warming.

Whilst previous seagrass studies in Gazi Bay have focused on species distribution, community composition, growth dynamics, nutrient content and carbon export between the seagrasses and the adjacent ecosystems [19–26], no study here (or elsewhere in Africa) has considered the carbon stocks and how these might compare with naturally occuring un-vegetated areas. The current work aimed to fill this gap by estimating the carbon stocks in the seagrass meadows of Gazi Bay, Kenya. At the same time, the work explored whether different seagrass C_org_ pools could be estimated using easy to measure parameters such as the above-ground biomass (AGB) and shoot height. Specifically, the objectives of the study were:

1. To compare sediment C_org_ between areas with seagrass and adjacent un-vegetated areas for each of the four dominant seagrass species in order to estimate the impact of these species on sediment carbon storage.
2. To determine the % C_org_ associated with each of the four dominant seagrass species at Gazi Bay and the relative contribution of biomass and sediment to the C_org_ per unit area of the seagrass species as well as their relationship to the above-ground parameters.
3. To explore the relationships between sediment C_org_ and the above ground carbon (and also above-and belowground biomass ratios for the dominant seagrass species of Gazi Bay.

## Materials and methods

### Study site

**Fig 1:**
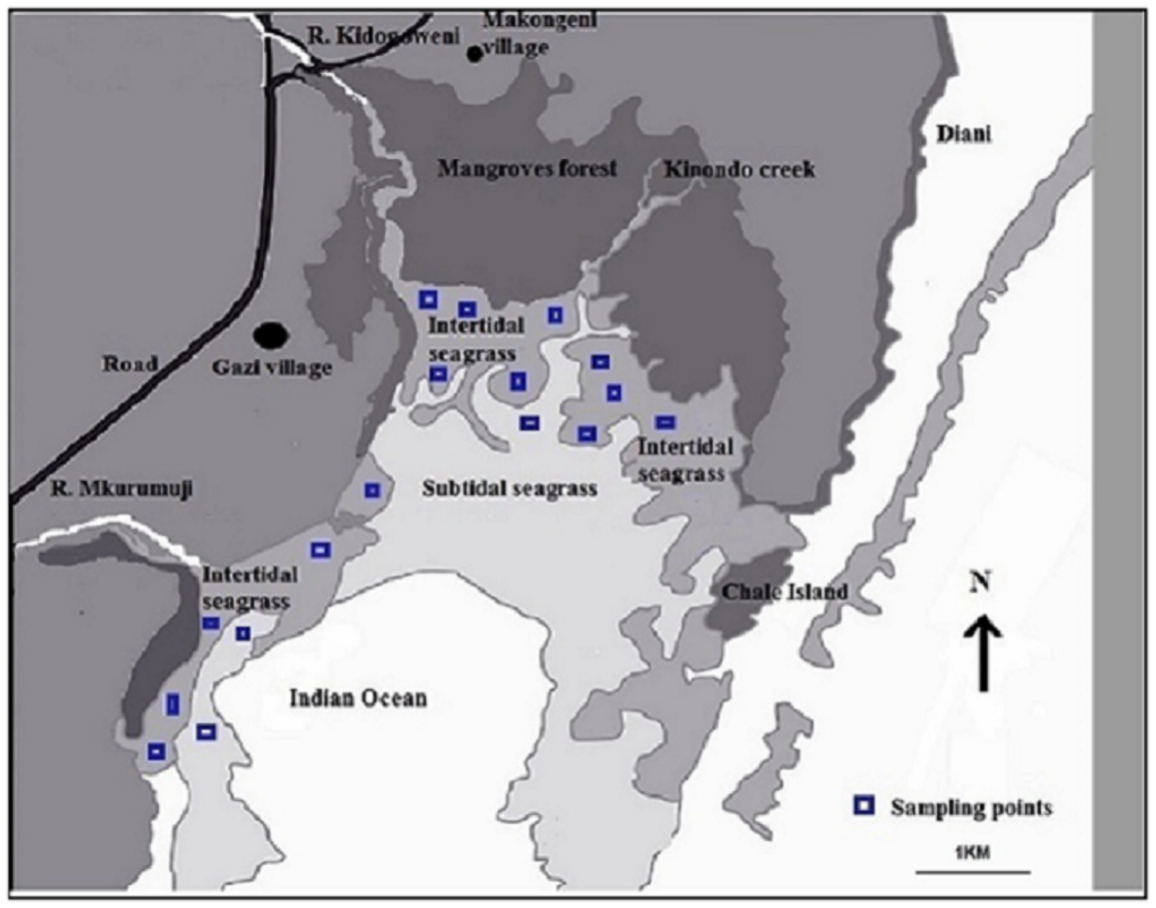
Seagrass sampling points in the seagrass meadows of Gazi Bay, Kenya.

This study was carried out at Gazi Bay (4°25’S, and 39°30’E), located on the southern coast of Kenya, about 55 km from Mombasa City through permission issued by the National Commission for Science, Technology & Innovation (NACOSTI): Permit no NACOSTI/P/14/2443/769 on 17^th^ February 2014. The bay is a shallow tropical coastal water system (mean depth < 5m), approximately 1.75–3.5 km wide and 3.25 km long with a surface area of ~ 17 km^2^ [22]. It is open to the Indian Ocean through an entrance in the south with depths varying between 3 and 8m in the eastern and western regions respectively [27]. It is characterized by two creeks, a western creek that extends to a fresh water inflow (R. Kidogoweni) on the north western side of the bay and an eastern creek that lacks such an inflow. On the south western side of the bay is another fresh water inflow from R. Mkurumuji (Fig 1).

The main climatic drivers are the southeast monsoons (May-September) and the northeast monsoons (November-March). An offshore wind prevails throughout the year. Long rains occur between March and May while the short rains occur between October and November [27,28]. However, inter-annual shifts in these seasons are common [29]. The tide at Gazi Bay is normally mixed semi-diurnal, with a tidal range of 1.4 m and 3 m at neaps and springs respectively. These tides generate strong reversing currents in the tidal creeks and relatively weaker currents in the open regions of the bay. The shoreward wind and the tidal currents combine to vertically mix the water column in the bay, leading to formation of a more homogeneous water, with a salinity range of 34.5–35.5ppt. A significant lateral and vertical salinity gradient develops during the rainy periods as a result of increased river discharge and the runoff [27]. The flushing ability of mixed semi-diurnal tides in the bay varies depending on tidal range, tidal elevation, and the nature of the tide. Tides are generally swift during springs and tend to rapidly disperse lower salinity water [27,30]. The rates of water exchange are also high during spring tides compared to neap tides. On the other hand, currents at neaps are sluggish and inhibit flushing of brackish water. This flushing pattern of the tide combined with river runoff has a far-reaching effect in the form of nutrient and material exchanges on the linkage between mangrove, seagrass, and coral reef ecosystems [30–32]. Seagrasses, which are found at the centre of the bay, cover an area of ~ 7 km^2^ [21,26,28].

All the twelve seagrass species described along the East African coast have been recorded in Gazi Bay, usually attached to both soft and hard substrates in the bay [33–35]. The seagrass community in the bay consists of four dominant species: *Thalassodendron ciliatum* (Forssk.) den Hartog, *Thalassia hemprichii* (Enhrenberg) Aschers., *Enhalus acoroides* (L.f.) Royle and *Syringodium isoetifolium* (Aschers.) Dandy. These are observed to grow either as monospecific stands or mixed with other seagrass species, with their coverage extending from the intertidal to the subtidal areas in sandy and rocky substrates [22]. The other, less abundant species, are: *Cymodocea rotundata* Ascherson, *Cymodocea serrulata* (R. Braun) Aschers. & Magnus, *Halodule uninervis* (Forssk.) Aschers., *Halodule wrightii* (Aschers.), *Halophila minor* (Zoll.) den Hartog, *Halophila ovalis* (Braun) Hooker, *Halophila stipulaceae* (Forssk.) Aschers. and *Zostera capensis* (Setch) [22,35]. The meadows are usually luxuriant for most of the year except during short periods of intense grazing and desiccation. The seagrass meadows of Gazi Bay fall under Diani-Chale Marine National Reserve which was established in 1994. It also forms the northern boundary of the proposed Transboundary Marine Conservation Area between Kenya and Tanzania (TBCA) [36]. At present there is no enforcement of its protected status due to weak governance. The use of seine and drag nets by artisanal fishermen is a daily activity in the shallow waters of the bay. Although there are no published data on the effects of this fishing, anecdotal information and personal observation suggests this is a cause of degradation of the seagrass in areas of intense fishing.

### Sampling design

We used sampling precedures specific for coastal blue carbon and the revised Intergovernmental Panel for Climate Change (IPCC) carbon accounting protocols for coastal wetlands [37,38]. Intensive studies were made within mono-specific stands of four seagrass species: *T*. *hemprichii*, *E*. *acoroides*, *T*. *ciliatum* and *S*. *isoetifolium*. These species were selected on the basis of their local dominance, as determined by the initital reconnaisance survey carried out by the team. Samples were taken from a wide area following a stratified random design, which extended from plots adjacent to the mangrove forest, all the way to the seagrasses near the mouth of the R. Mkurumuji (Fig 1), in order to sample the spatial variability of biomass and sediment C_org._ associated with the four target species in the bay. Forty quadrats, each measuring 0.25 m^2^, were sampled for the biomass carbon for each of the three species, *T*. *hemprichii*, *E*. *acoroides* and *T*. *ciliatum* whilst 20 quadrats were sampled for *S*. *isoetifolium*, owing to its relatively small spatial distribution.

### Determination of species type, canopy cover, shoot density and canopy height

Sample plots were established within the identified seagrass strata placing the quadrats of 0.25m^2^ at minimum intervals of 15m in each seagrass strata. This was done during low spring tides since at this time the seagrass beds are exposed and accessible on foot. Species type was determined *in situ* with the help of field manuals [39]. Shoot density and canopy cover were determined by counting all the shoots within quadrats and extrapolating to per m^2^ while the % canopy cover was obtained through visual estimates. Canopy height was determined through the measurement of heights of 10% of randomly selected individual shoots from the total within the quadrat and calculating the mean heights.

### Estimation of above-ground and below-ground biomass

The above-ground biomass (AGB) was obtained by harvesting all plant materials above-ground within the 0.25m^2^ quadrats. In the laboratory, the seagrass was cleaned with fresh water, sorted and scraped with a razor blade to remove epiphytes. Seagrass fronds were then washed in 10% hydrochloric acid to remove any calcareous material after which they were dried in an oven at 60°C for 72 hours. For below-ground biomass, four cores were taken in each of the four quarters of the 0.25m^2^ quadrats with a Russian peat sampler (50cm long and 5cm diameter). Initial washing was done in the field using a 500μm sieve. In the laboratory further washing and rinsing of the samples was carried out. Upon sieving, the materials were sorted into component parts: roots, rhizomes and necromass (that is dead roots or rhizomes) and dried in an oven at 60°C for 72 hours. The combined below-ground biomass values for the roots, rhizomes and necromass from the four cores per quadrat were summed and then converted to per m^-2^. The total biomass carbon was obtained by multiplying biomass with a carbon conversion factor of 0.34 (assuming that carbon constitutes 34% of the biomass) and then extrapolated to per hectare following recommended protocols for estimating carbon for marine ecosystems [37,38].

### Measurements of bulk density, % organic matter and sediment C_org_

Two sediment cores, each extending to a depth of 50cm, were collected in each quadrat, using the peat sampler from the vegetated and the un-vegetated areas, chosen to act as natural ‘controls’ for each species. Vegetated areas were identified as those with seagrass cover while un-vegetated areas were the naturally occurring bare patches, measuring 3 to 6m in diameter, in the midst of the seagrass covered areas.

In the laboratory, the samples were sliced into 5cm sub-sections and were oven dried at 60°C for 72 hours to obtain a constant weight. Similar cores were collected in un-vegetated seagrass areas to serve as ‘controls’.

Dry bulk density (DBD) (the dry weight of sediment per unit volume) was calculated for each of the ten sub-sections per core as follows:

$$\text{DBD }\left(\text{g}/{\text{cm}}^{3}\right)=\text{Dry weight}/\text{Original volume of the sediment}$$

Organic matter was measured in each of the ten sub-sections per core by Loss on Ignition (LOI) techniques, using a muffle furnace at 450°C for 6 hours. LOI weight was used to calculate the %OM content as follows:

% LOI=((Initial dry weight–Weight remaining after ignition)/Initial dry weight)×100.

The sediment C_org_ values were arrived at using one of the two equations depending on the organic matter content of the sediment sample:

$$\%\text{ LOI }<\;0.2:\;\%{\text{ C}}_{\text{org}}=0.40*\%\text{ LOI}-0.21$$

$$\%\text{ LOI }>\;0.2:\;\%{\text{ C}}_{\text{org}}=0.43*\%\text{ LOI }-\;0.33,$$

following recommended protocols for estimating carbon for the marine ecosystems [3,37]. Estimates for the top 50 cm of the sediment were extrapolated to one metre.

### Data analysis

Assumptions of normality and homogeneity of variance were tested by examining residuals in all relevant tests; where these were not met, data were transformed to meet parametric assumptions. One way ANOVAs were used to test for the differences in above-ground, below-ground and total biomass between species, with Tukey *post—hoc* analyses used to compare means when significant differences were detected. Linear regression analysis was used to determine possible relationships between above-and below-ground biomass and also between sediment C_org_ and the above-ground biomass for the species areas. Nested two-way ANOVAs were used to compare sediment C_org_ between each species and its un-vegetated control cores. In all statistical tests, the significance level was set at α = 0.05.

## Results

### Sediment C_org_ in seagrass areas and un-vegetated ‘controls’

**Fig 2:**
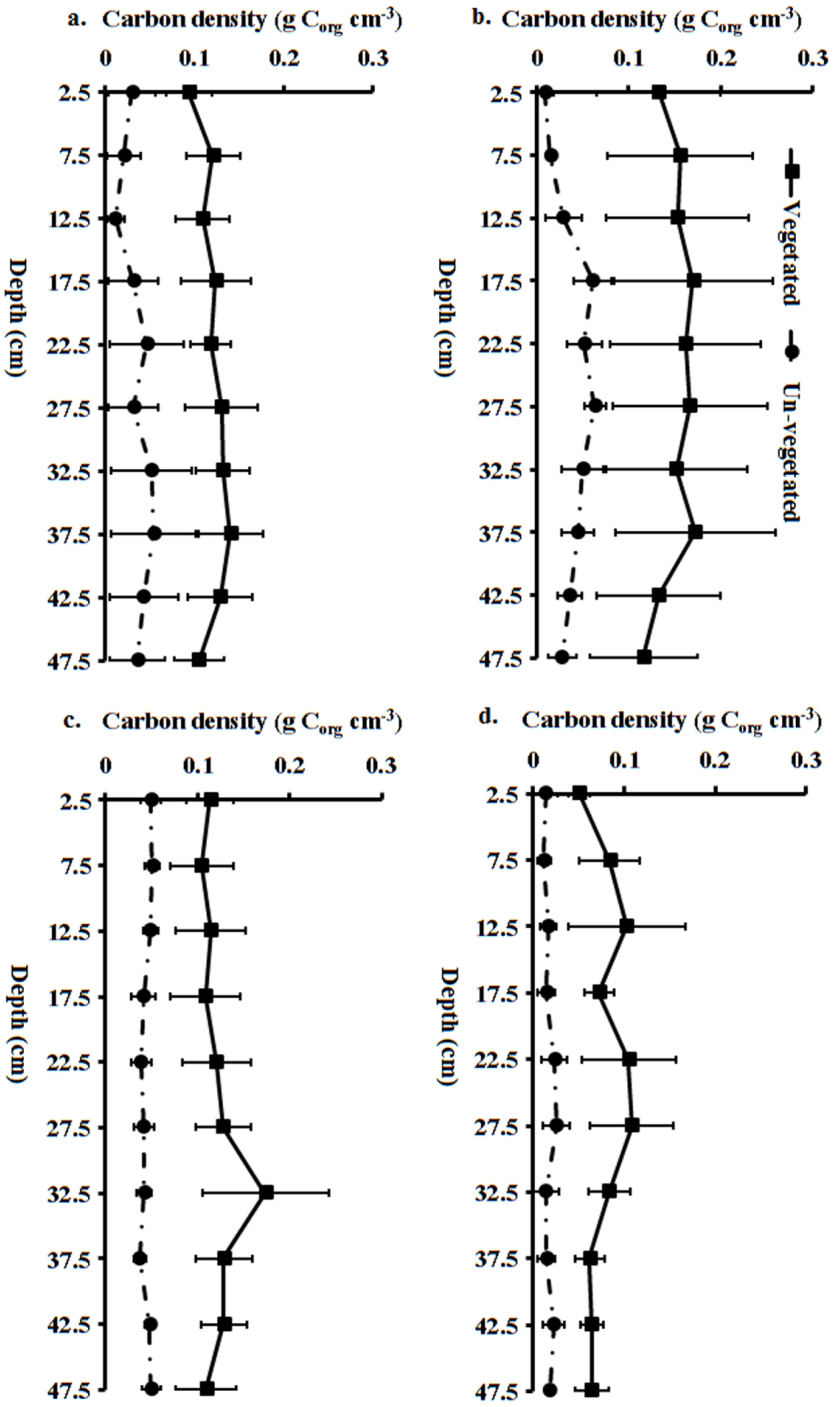
Carbon density (mean ± 95% C.I.) along depth profiles in the vegetated and un-vegetated areas associated with the dominant seagrass species of Gazi Bay (a. *T*. *hemprichii* b. *E*. *acoroides* c. *T*. *ciliatum* d. *S*. *isoetifolium*).

There was higher sediment C_org_ in the vegetated seagrass areas compared to the un-vegetated areas (Fig 2). Strikingly these differences persisted down to 50cm depth in all species; initial analyses using depth and ‘treatment’ (i.e. vegetated vs un-vegetated) as fixed factors in two way ANOVAs showed significant treatment effects but no depth or depth-treatment interaction effects. Hence fully nested ANOVAs, in which depth was nested within cores and treatment (vegetated/un-vegetated) to recognise non-independence of depth slices from the same cores, were subsequently used. These revealed highly significant effects of treatment on C_org_ density for each of the species: F _(1, 180)_ = 38.68, *p* < 0.001 for *T*. *hemprichii*; F _(1, 180)_ = 27.89, *p* < 0.001 for *T*. *ciliatum*; F _(1, 180)_ = 32.16; *p* < 0.001 for *E*. *acoroides* and F _(1, 180)_ = 11.55, *p* = 0.003 for *S*. *isoetifolium*.

**Fig 3:**
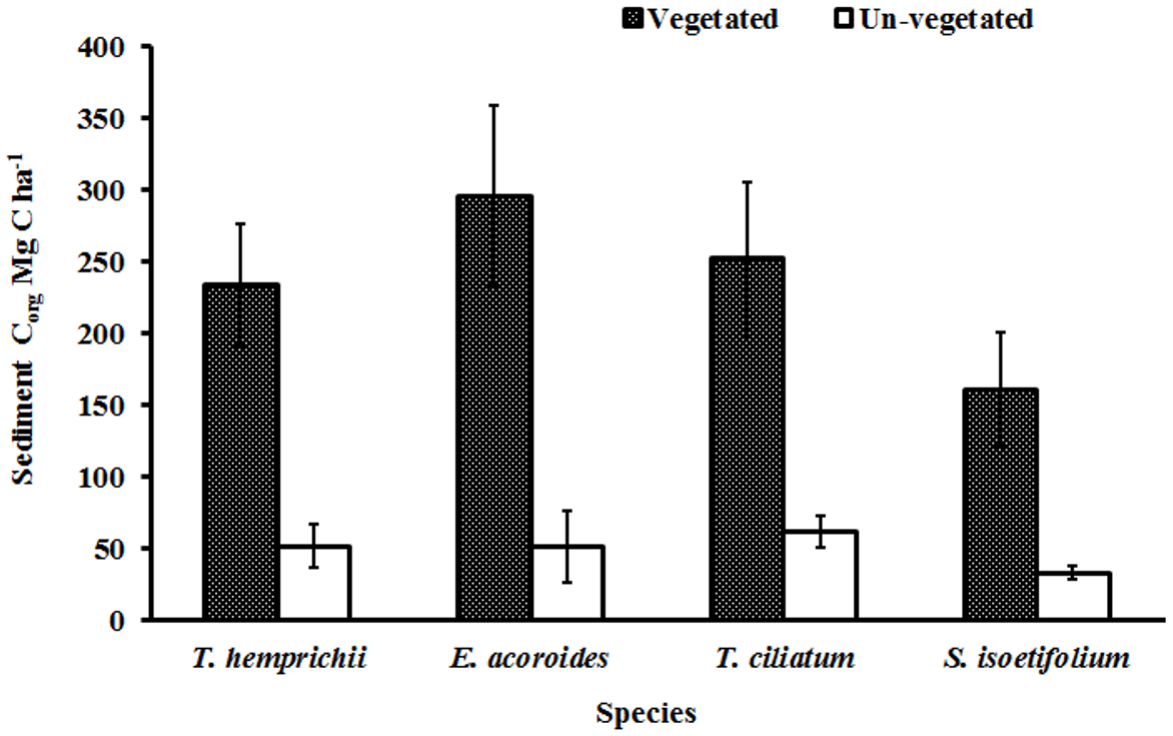
Variation in sediment C_org_ between the vegetated and un-vegetated areas for the four seagrass species (means± 95% C.I.).

The sediment C_org_ varied between meadows of different species with the highest being recorded in *E*. *acoroides* at 295.7 ± 63.6 (mean ± 95% C.I) Mg C ha^-1^ and the lowest in *S*. *isoetifolium* at 160.7 ± 40.3 Mg C ha^-1^ (Fig 3). A one way ANOVA showed significant differences in C_org_ among the species (F _(3, 56)_ = 6.24, *p* = 0.001).

### Comparison of the sediment C_org_ and the above-ground biomass C_org_

**Fig 4:**
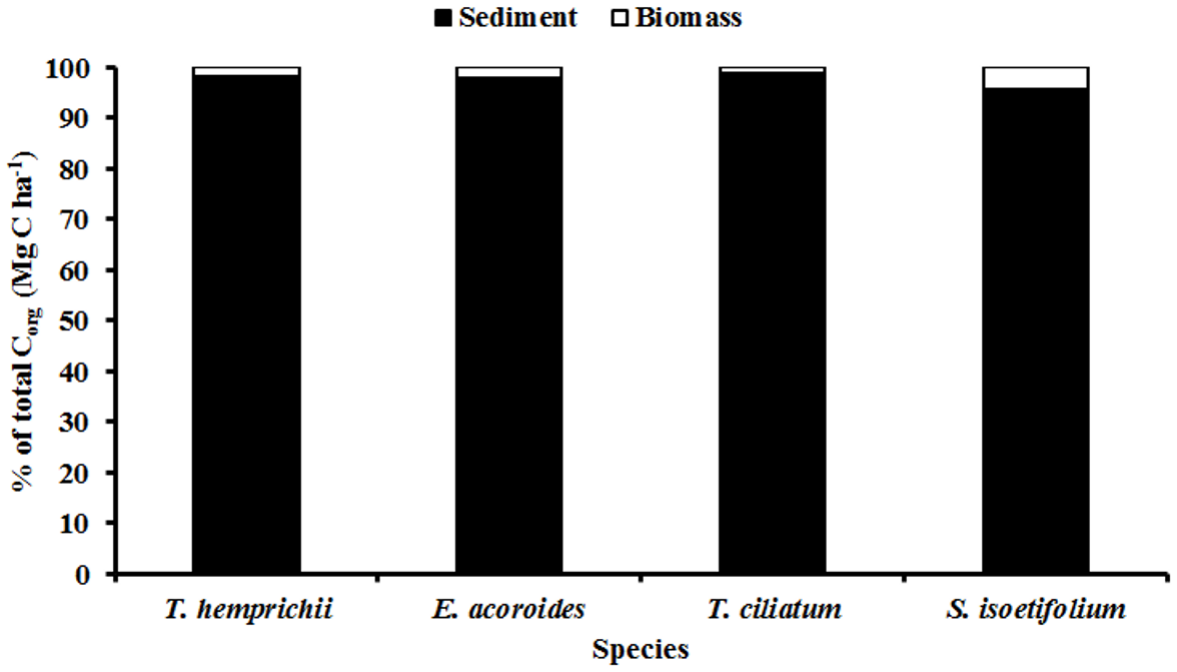
Relative % of the total C_org_ (± 95% C.I) for the sediment and the biomass associated with the four dominant seagrass species at Gazi Bay.

The sediment C_org_ constituted the bulk of the total C_org_, in all four species areas (Fig 4). The sediment C_org_ of 295.7 ± 63.6 Mg C ha^-1^ (mean ± 95% C.I) for *T*.*ciliatum* accounted for the highest proportion at 98.1% of the total C_org_ per unit area dominated by the species while 95.8% was the lowest proportion recorded in *S*. *isoetifolium* areas. Plotting the sediment C_org_ against the above-ground biomass C_org_ for each of the four seagrass species showed no significant relationships.

### Relationships between above-and below-ground biomass

The highest mean AGB was associated with *T*. *ciliatum* while the lowest was associated with *E*. *acoroides* (Table 1). The highest mean BGB was recorded in the *S*. *isoetifolium* while the lowest was recorded in the *T*. *ciliatum*. Comparison of AGB and BGB relationships in the four species revealed a highly significant relationship in *E*. *acoroides*, (F _(1, 38)_ = 25.02, *p* < 0.001) but there were no significant relationships between the BG and the AGB in the other species. BGB constituted the highest biomass component with an average of 82.2 ± 8% (or ~ 4:1 BG:AG biomass ratio) for all the species, with *E*. *acoroides* having the highest at 90.9 ± 1% and *T*. *ciliatum* having the lowest at 71.0 ± 5%.

**Table 1:** Mean (± 95% C.I) shoot density, canopy cover (%), canopy height (cm), above-ground (AGB), below-ground (BGB) and total biomass (TB) characteristics of the dominant seagrass species at Gazi Bay, Kenya.

Species	Shoot density(m^-2^)	% C. cover	C. ht (cm)	AGB (g. DW m^-2)^	BGB g. DW m^-2^)	TB (g. DW m^-2^)	% BGB
*T*. *hemprichii*	996 ± 94	69.3 ± 4.2	18.4 ± 1.4	202.1 ± 29.9	1361.1 ± 281.8	1563.1± 279.2	82.8±3
*E*. *acoroides*	248 ± 28	47.5 ± 4.2	55.1 ±4 1	155.9 ± 23.7	1669.2 ± 217.6	1825.8 ± 234.8	90.9±1
*T*. *ciliatum*	531 ± 67	61.9 ± 3.7	36.7 ±3.9	308.2 ± 33.5	1096.6 ± 221.4	1404.9 ± 232.5	71.0±5
*S*. *isoetifolium*	4351 ± 500	72.0 ± 7.0	23.3 ± 2.7	300.8 ± 42.6	1683.8 ± 242.9	1984.7 ± 245.5	84.0±4

### Relationships between the total biomass and above-ground parameters

Total biomass varied between the species with the highest being recorded in *S*. *isoetifolium* at 1985 ± 246 g DW m^-2^ while *T*. *ciliatum* had the lowest at 1405 ± 233 g DW m^-2^. The total biomass was significantly different between the species (F _(3, 136)_ = 4.13, *p* = 0.008). No significant relationships were found between the total biomass and shoot density for any of the species and neither between the total biomass and the shoot height, except in *T*. *ciliatum* (F_(1,38)_ = 9.83, *p* = 0.004). The positive relationship between shoot density and % canopy cover was apparent across species (Table 1).

## Discussion

The current study investigated biomass and sediment C_org_ stocks associated with the four dominant seagrass species of Gazi Bay, providing amongst the first data from Africa for seagrass sediment C_org_ [40]. It also compared sediment C_org_ in contiguous vegetated and un-vegetated areas for each of the species and demonstrated highly significant differences in carbon density, with the presence of seagrass enhancing sediment carbon stocks by a factor of 4–6, depending on the species. The mean sediment C_org_ estimated for the top one metre of the sediment (using IPCC protocols to extrapolate downwards from the 50cm measured depth) from seagrass vegetated areas was 236 ± 24 Mg C ha^-1^. This is well above the mean of 166 Mg C ha^-1^ derived from a global data set [3], (although within the range of 115.5–829.2 Mg C ha^-1^). Across all species, sediment depth did not have a significant effect on sediment C_org_ and the differences observed between vegetated and un-vegetated areas were consistent down to 50cm. This suggests a surprising degree of spatial consistency and longevity in these relatively small (typically 3 to 6m diameter) patches of seagrass meadow and bare areas. Sediment C_org_ dominated the carbon stocks in all species areas, constituting over 97% of the total C_org_ compared to less than 3% contributed by the biomass. Much of this sediment carbon is likely to be allocthonous; globally an average of ~50% of sediment C_org_ associated with seagrass meadows is derived from external sources [4]. In Gazi Bay much of this comes from mangroves; previous studies in Gazi Bay showed that mangrove carbon is exported to the seagrass beds where it is stripped from the water column and settles [25,41] with an estimated 21–71% of carbon exported from the fringing mangrove forests captured within the seagrass meadows [22].

Whilst there is a growing literature exploring sediment carbon in seagrass meadows there are few explicit comparisons of vegetated with un-vegetated areas. A limited number of studies have compared the sediment carbon stocks or both the stocks and burial rates in bare areas and naturally or artificially recolonized meadows [14,40–45]. For instance, a study on carbon accumulation in a restored seagrass meadow in Virginia, USA, in which sediment cores were taken to 10-20cm depth, [44] reported that after 9 years the meadow had 3 times more carbon than the un-vegetated areas, suggesting powerful effects of seagrass on C sequestration at this site and a rapid recovery in this service following restoration. A synthesis of global data [5] gave a mean sediment C burial rate of 138 g C m^-2^ yr^-1^ in seagrass beds. A recent study in Oyster Harbour of Western Australia [40] reported that a restored seagrass meadow took 18 years to acquire a carbon accumulation rate of 26.4 ±0.8 g C m^-2^ yr^-1^ and that a naturally vegetated area which was used as a reference had 63% and 37% more carbon than the un-vegetated and restored areas respectively. To our knowledge, the present study is the first one to compare sediment C_org_ for seagrass in naturally occuring vegetated and un-vegetated areas in Africa and it demonstrates an exceptionally powerful effect of seagrass on C sequestration, with relative C densities being higher in areas under seagrass compared with appropriate un-vegetated control areas and the differences being higher than in any other relevant studies.

A recent study using a global data set [46] reported an average sediment accretion rate of 2mm yr^-1^ for seagrasses. If the sediment at Gazi accumulated at the same rate this suggests a minimum age of 250 years for sediment at 50cm, demonstrating that the current locations of seagrass meadow and bare patches are likely to have persisted for at least decades. However, a robust estimation method requires in-situ dating studies.

The present study did not establish any significant relationships between sediment and biomass C_org_ in any of the species. Lack of strong relationships between either the sediment C_org_ and the AGB or between the AGB and BGB measures means that AGB was not a suitable proxy for the determination of BGB or sediment C_org_ for any of these species and coring is therefore necessary for accurate estimates of these variables.

The biomass estimates obtained in the present study were within the range of published data for the same species in other parts of the world although tended towards the high end of these ranges [18,34,47]. The mean for the total biomass C_org_ for the four seagrass species was 5.9 ± 0.9 Mg C ha^-1^. As was the case for sediment C_org,_ this value is well above the global mean of 2.51 ± 0.49 Mg C ha^-1^ (although *Posidonia oceanica* in the Mediteranean has greater biomass than our species, with a mean of 7.29 ± 1.52 Mg C ha^-1^) [3]. Across all species, the BGB was much higher than the AGB, accounting for over 80% of the total. The fact that AGB in all species was substantially lower and slightly more variable than the BGB could be attributed to higher turnover rates for the AGB occasioned by grazing pressure, mechanical removal by tides and human activities such as seine fishing, events that were observed in the bay in the course of this study. Whilst larger species, such as *E*. *acoroides*, with large fronds and big roots and rhizomes, are likely to accumulate more biomass during growth which is invested in their below-ground tissues, shoot density is also an important parameter in determining per unit area BGB. *Syringodium isoetifolium*, which had a substantially higher shoot density and % canopy cover than the other species, recorded higher BGB than even the larger species.

Seagrasses cover an estimated area of 7km^2^ within Gazi Bay [22], this is approximately 41% of the bay area considering the entire bay area of 17km^2^). Extrapolation of the mean biomass and the sediment C_org_ values in the top one metre of the sediment gives an estimated total of 168,642 Mg C for seagrass meadow carbon stocks in the bay as a whole. If these findings are representative of seagrass meadows along much of the African coast the current absence of African sites from the global data will result in an underestimate of average carbon storage in seagrass meadows.

There are many sources of uncertainty in this estimate, however; for example we considered only the four dominant species, which constitute ~ 70% of the seagrasses of the bay, and other species areas may show different C densities. Future research at Gazi should not only aim to quantify the C_org_ from all seagrass species but should undertake a thorough mapping and estimation of the sedimentary C_org_ of the seagrasses of the entire bay for a better understanding of the carbon storage capacity of seagrasses there.

The present study has established that, as for other species and sites, the sediment C_org_ constitutes by far the major C pool for the seagrass beds of Gazi Bay. The highly significant differences in C_org_ between vegetated and un-vegetated areas underlines the importance of seagrass meadows as shallow marine C sinks, a service that adds to the many other justifications for their conservation. This study provides among the first estimates of sediment C_org_ from seagrasses in the Africa. As such it contributes to the growing global literature on the importance of seagrass meadows as C sinks. It also provides information of potential relevance to the conservation and management of seagrasses in the area. Gazi Bay hosts a pioneer carbon offset project “Mikoko Pamoja”, the first initiative in the world to restore and protect mangroves through the sale of carbon credits (http://www.planvivo.org/project-network/mikoko-pamoja-kenya). Knowledge of the carbon stocks associated with seagrasses in the bay may open opportunities for bundling seagrass ecosystem services with those of the mangrove ecosystem, an approach that makes ecological sense, given the strong connections between the two ecosystems.

## Supporting information

S1 File Carbon density (mean ± 95% C.I.) along depth profiles in the vegetated and un-vegetated areas associated with the dominant seagrass species of Gazi Bay (a. *T*. *hemprichii* b. *E*. *acoroides* c. *T*. *ciliatum* d. *S*. *isoetifolium*). (XLSX)

S2 File Variation in sediment C_org_ between the vegetated and un-vegetated areas for the four seagrass species (means± 95% C.I.). (XLSX)

S3 File Relative % of the total C_org_ (± 95% C.I) for the sediment and the biomass associated with the four dominant seagrass species at Gazi Bay. (XLSX)

## References

[1] Nellemann C, Corcoran E, Duarte C, Valdes L, De Young C, Fonseca L, et al Blue carbon A Rapid Response Assessment (78) United Nations Environmental Programme, GRID-ARENDAL 2009.

[2] Lavery PS, Mateo M-Á, Serrano O, Rozaimi M. Variability in the carbon storage of seagrass habitats and its implications for global estimates of blue carbon ecosystem service. PLoS One. 2013;8(9):e73748 10.1371/journal.pone.0073748 24040052 PMC3764034

[3] Fourqurean JW, Duarte CM, Kennedy H, Marbà N, Holmer M, Mateo MA, et al Seagrass ecosystems as a globally significant carbon stock. Nat Geosci. Nature; 2012;5(6):1–5.

[4] Kennedy H, Beggins J, Duarte CM, Fourqurean JW, Holmer M, Marbá N, et al Seagrass sediments as a global carbon sink: Isotopic constraints. Global Biogeochem Cycles. 2010;24(4).

[5] Mcleod E, Chmura GL, Bouillon S, Salm R, Björk M, Duarte CM, et al A blueprint for blue carbon: toward an improved understanding of the role of vegetated coastal habitats in sequestering CO_2_. Front Ecol Environ. 2011;9(10):552–60.

[6] Duarte CM, Cebrián J. The fate of marine autotrophic production. Limnol Oceanogr. 1996;41(8):1758–66.

[7] Duarte CM, Middelburg JJ, Caraco N. Major role of marine vegetation on the oceanic carbon cycle. Biogeosciences. 2005;2:1–8.

[8] Watson R A., Coles RG, Long WJL. Simulation estimates of annual yield and landed value for commercial penaeid prawns from a tropical seagrass habitat, northern Queensland, Australia. Aust J Mar Freshw Res. 1993;44(1990):211–9.

[9] De La Torre-Castro M, Ronnback P. Links between humans and seagrasses—An example from tropical East Africa. In: Ocean and Coastal Management. 2004 p. 361–87.

[10] Orth RJ, Carruthers TJB, Dennison WC, Duarte CM, Fourqurean JW, Heck KL, et al A Global Crisis for Seagrass Ecosystems. Bioscience. 2006;56(12):987–97.

[11] Romero J, Pe’rez M, Mateo MA, Sala E. The belowground organs of the Mediterranean seagrass *Posidonia oceanica* as a biogeochemical sink. Aquat Bot. 1994;47(1):13–9.

[12] McGlathery Karenck K, Anderson IC. Eutrophication in shallow coastal bays and lagoons: The role of plants in the coastal filter. Vol. 348, Marine Ecology Progress Series. 2007 p. 1–18.

[13] Costanza R, Arge R, De Groot R, Farberk S, Grasso M, Hannon B, et al The value of the world’s ecosystem services and natural capital. Nature. 1997;387:253–60.

[14] Macreadie PI, York PH, Sherman CDH, Keough MJ, Ross DJ, Ricart AM, et al No detectable impact of small-scale disturbances on “blue carbon” within seagrass beds. Mar Biol. 2014;161(12):2939–44.

[15] Chmura GL, Anisfeld SC, Cahoon DR, Lynch JC. Global carbon sequestration in tidal, saline wetland soils. Global Biogeochem Cycles. 2003;17(4):12.

[16] Gacia E. Sediment Retention by a Mediterranean *Posidonia oceanica* Meadow: The Balance between Deposition and Resuspension. Estuar Coast Shelf Sci. 2001;52(4):505–14.

[17] Marba’ N, Hemminga MA, Mateo MA, Duarte CM, Mass YEM, Terrados J, et al Carbon and nitrogen translocation between seagrass ramets. Mar Ecol Prog Ser. 2002;226:287–300.

[18] Duarte Carlos M, Chiscano Carina L. Seagrass biomass and production: a reassessment. Aquat Bot. 1999;65:159–74.

[19] Githaiga MN, Gilpin L, Kairo JG, Huxham M. Biomass and productivity of seagrasses in Africa. Bot Mar. 2016;59(2–3):173–86.

[20] Waycott M, Duarte CM, Carruthers TJB, Orth RJ, Dennison WC, Olyarnik S, et al Accelerating loss of seagrasses across the globe threatens coastal ecosystems. Ecology. 2009;106(30):12377–81. 10.1073/pnas.0905620106 PMC2707273 19587236

[21] Pendleton L, Donato DC, Murray BC, Crooks S, Jenkins WA, Sifleet S, et al Estimating Global “Blue Carbon” Emissions from Conversion and Degradation of Vegetated Coastal Ecosystems. PLoS One. 2012;7(9). 10.1371/journal.pone.0043542 PMC3433453 22962585

[22] Bouillon S, Dehairs F, Velimirov B, Abril G, Borges A. Dynamics of organic and inorganic carbon across contiguous mangrove and seagrass systems (Gazi Bay, Kenya). J Geophys Res. 2007;112(G2):G02018.

[23] Coppejans EH, Beeckman De Witt M. The seagrass and associated macroalgae vegetation of Gazi Bay (Kenya). Hydrobiologia. 1992;247:59–75.

[24] Duarte CM. Growth and population dynamics of *Thalassodendron ciliatum* in a Kenyan back-reef lagoon. Aquat Bot. 1996;55:1–11.

[25] Hemminga MA, Slim FJ, Kazungu J, Ganssen GM, Nieuwenhuize J, Kruyt NM. Carbon outwelling from a mangrove forest with adjacent seagrass beds and coral reefs (Gazi Bay, Kenya). Mar Ecol Prog Ser. 1994;106(3):291–302.

[26] Hemminga MA, Gwada P, Slim FJ, de Koeyer P, Kazungu J. Leaf production and nutrient contents of the seagrass *Thalassodendron ciliatum* in the proximity of a mangrove forest (Gazi Bay, Kenya). Aquat Bot. 1995;50(2):159–70.

[27] Kitheka JU. Water circulation and coastal trapping of brackish water in a tropical mangrove-dominated bay in Kenya. Limnol Oceanogr. 1996;41(l):169–76.

[28] Schott FA, Xie S-P, McCreary JP. Indian Ocean Circulation and Climate Variability. Rev Geophys. 2009;47:1–46.

[29] Haas LW. The Effect of the Spring-neap Tidal Cycle on the Vertical Salinity Structure of the Analysis of salinity data collected during the period following Tropical. Estuar Coast Mar Sci. 1977;5:485–96.

[30] Prrez-llorrns JL. Seasonal dynamics of biomass and nutrient content in the intertidal seagrass *Zostera noltii* Hornem from Palmones River estuary, Spain. 1993;46.

[31] Ho CL, Barrett BB. Distribution of Nutrients in Louisiana’s Coastal Waters Influenced by the Mississippi River and. 1976;(1967):173–95.

[32] Dahdouh E, D Van Speybroeck. Remote sensing and zonation of seagrasses and algae along the Kenyan coast. Hydrobiologia. 1999;400:63–73.

[33] Bandeira SO. Leaf production rates of *Thalassodendron ciliatum* from rocky and sandy habitats. Aquat Bot. 2002;72(1):13–24.

[34] Erftemeier P. Factors limiting growth and production of tropical seagrasses: Nutrient dynamics in Indonesian seagrass beds. Catholic University of Nijmegen. The Netherlands; PhD Thesis, 1993.

[35] Isaac W, Isaac F. Marine botany of the Kenya coast. General account of the environment, flora and vegetation. JE Afr Nat Hist Soc. 1968;27(116):7–27.

[36] MPRUT-KWS. A proposed marine Transboundary Conservation Area between Kenya and Tanzania, 71pp, 2016.

[37] Howard J, Hoyt S, Isensee K, Telszewski M and Pidgeon E (eds,). Coastal Blue Carbon:Methods for assessing carbon stocks and emmission factors in mangroves, tidal salt marshes and seagrasses. Conservation International, Intergovernmental Oceanographic Commission of UNESCO, International Union for Conservation of Nature. In: Conservation International Arlington, Virginia, USA.; 2014 p. 99–107.

[38] IPCC. (2014) 2013 Supplement to the 2006 IPCC guidelines for national green house gas inventory, Wetlands. Vol. 2. 2013.

[39] Richmond MD (ed.). A Guide to the Seashores of Eastern Africa and the Western Indian Ocean Islands. Sweden: Sida/Department for Research Cooperation, SAREC 448pp. 1997.

[40] Dahl M, Deyanova D, Lyimo LD, Johan N, Gullstr M. Effects of shading and simulated grazing on carbon sequestration in a tropical seagrass meadow. J Ecol. 2016;104:654–64.

[41] Bouillon S, Moens T, Dehairs F. Carbon sources supporting benthic mineralization in mangrove and adjacent seagrass sediments (Gazi Bay, Kenya). Biogeosciences. 2004;1(1):71–8.

[42] Marba’ N, Arias-Ortiz A, Masque P, Kendrick GA, Mazarrasa I, Bastyan GR, et al Impact of seagrass loss and subsequent revegetation on carbon sequestration and stocks. J Ecol. 2015;103(2):296–302.

[43] Serrano O, Lavery PS, López-Merino L, Ballesteros E, Mateo MA. Location and Associated Carbon Storage of Erosional Escarpments of Seagrass *Posidonia Mats*. Front Mar Sci. 2016;3:42.

[44] Serrano O, Ruhon R, Lavery PS, Kendrick GA, Hickey S, Masqué P, et al Impact of mooring activities on carbon stocks in seagrass meadows. Sci Rep. Nature; 2016;6:23193. 10.1038/srep23193 PMC4793266 26979407

[45] Irving AD, Connell SD, Russell BD. Restoring coastal plants to improve global carbon storage: reaping what we sow. PLoS One. 2011;6(3):e18311 10.1371/journal.pone.0018311 21479244 PMC3066232

[46] Greiner JT, McGlathery KJ, Gunnell J, McKee B a. Seagrass Restoration Enhances “Blue Carbon” Sequestration in Coastal Waters. PLoS One. 2013;8(8):e72469 10.1371/journal.pone.0072469 23967303 PMC3743776

[47] Duarte CM, Losada IJ, Hendriks IE, Mazarrasa I, Marbà N. The role of coastal plant communities for climate change mitigation and adaptation. Nat Publ Gr. Nature; 2013;3(11):961–8.

